# Sport injuries in international masters rowers: a cross-sectional study

**DOI:** 10.3325/cmj.2018.59.258

**Published:** 2018-10

**Authors:** Tomislav Smoljanović, Ivan Bohaček, Jo Hannafin, Henning Bay Nielsen, Darko Hren, Ivan Bojanić

**Affiliations:** 1 Department of Orthopedic Surgery, University Hospital Center Zagreb, University of Zagreb School of Medicine, Zagreb, Croatia; 2 Hospital for Special Surgery, Weill Medical College of Cornell University New York, Sports Medicine and Shoulder Service, New York City, NY, USA; 3 Department of Anesthesiology, Abdominal Centre, Rigshospitalet, Copenhagen, Denmark; 4 Chair for Psychology, Faculty of Humanities and Social Sciences, University of Split, Split, Croatia

## Abstract

**Methods:**

A cross-sectional study was conducted among 743 masters rowers who participated in the 34th International Federation of Rowing Associations (*Fédération Internationale des Sociétés d’Aviron*, FISA) World Rowing Masters Regatta held in Zagreb, September 2-9, 2007. A rowing-specific questionnaire was used, followed by an interview about the injuries sustained during the 12-month period before the competition.

**Results:**

The mean injury rate per year was 0.48 injuries/masters rower (2.25 injuries/1000 training sessions/rower). The majority of injuries were chronic injuries (the ratio of acute to chronic injuries was 1:1.7), and did not lead to the loss of training/competition time. Of all acute injuries, 49.6% were acquired during rowing-specific training, 43.7% during cross-training, and 6.7% in the gym. The most commonly affected region was the low back (32.6%), followed by the knee (14.2%), shoulder/upper arm, and elbow (10.6% each).

**Conclusion:**

International masters rowers sustained predominantly chronic injuries of low severity, and the most commonly injured region was the low back. The mean injury rate per rower per year was lower than the rates previously reported for juniors and seniors.

Participation in masters sports helps middle-aged and older adults to optimize their general health status, anthropometry, and physical function (1). Different masters categories in various sports include a wide variety of age subgroups, allowing fair competition between competitors/crews of similar age. In rowing, crew age categories are defined in the International Federation of Rowing Associations – *Fédération Internationale des Sociétés d’Aviron* (FISA) Rule Book (2). Rowers may start to compete in masters category from the beginning of the year in which they reach the age of 27. There are 13 crew age categories depending on the crew average age, where rowers' “age” is defined as the age the rower reaches during the year of the event (2). The categories are marked with letters A-M, with M being the category with average crew age of 89 years and older.

Rowing belongs to the group of Olympic sports with the lowest proportion of injured athletes (3-5). Elite level junior and senior international rowers sustain predominantly chronic (overuse) injuries (6,7). Although masters outnumber youth and elite rowers, there is no published data describing the injury incidence in this group (8-11). Rowing-specific studies and case reports of different medical problems among masters (12-16) are helpful in education of physicians but are lacking in incidence data.

The aim of our study was to estimate the frequency and location of musculoskeletal injuries in masters rowers according to age subgroups and to determine if there is a connection with different training modalities, rowing experience, and previous competition level.

## PARTICIPANTS AND METHODS

### Study design and data collection

This is a cross-sectional survey based on a rowing-specific four-page questionnaire, followed by an interview with rowers, conducted and designed as previously described (6,7). The survey included a group of masters rowers who participated at the 34th FISA World Rowing Masters Regatta held in Zagreb, Croatia, September 2-9, 2007.

The questionnaire was available in 24 different languages and consisted of three major sections, including personal and general rowing information; rowing information from the current season (from October 1, 2006 until the date of survey completion); and information about rowing injuries in that season (6,7). After having completed the questionnaire, the rowers were interviewed to obtain specific details regarding injuries sustained in the previous rowing season.

Injuries were divided according to the anatomic location and differentiated as either acute or chronic (17). Injuries were additionally subdivided based on the loss of training time, using previously published criteria (18) as follows: “incidental” injuries: no absence from competition or training; “minor”: absence shorter than one week; “moderate”: absence longer than one week, but shorter than one month; and “major”: absence from competition or training longer than one month.

### Participants

According to the available official data provided by the organizer of the regatta, the total number of competitors was 2910, representing 39 countries. We invited a convenient sample of 1000 master competitors (coxswains excluded) to complete the questionnaire. A total of 743 rowers (74.3% of the invited and 25.5% of the total number of competitors) from 36 countries completed and returned the questionnaires and were interviewed. The statistical analysis based on so many age categories would be difficult to perform and would probably yield no obvious conclusions on differences between age groups. Therefore, we divided rowers into three larger age groups as follows: 27-42-year age group (AB group), 43-59-year age group (CDE group), and 60 years and older age group (F+ group). There were 208 competitors in the AB group, 368 in the CDE group, and 167 in the F+ group (Table 1).

**Table 1:** Participants' general information*

	AB			CDE			F+			All		
Gender	men	women	both	men	women	both	men	women	both	men	women	both
Number	115	93	208	226	142	368	134	33	167	475	268	743
Age (median, Q; years)	36 (10)	35 (10)	35 (10)	51 (8)	50 (7)	50 (7)	66 (6)	67 (9)	66 (6)	51 (17)	46 (14)	50 (17)
Height (median, Q; cm)	187 (8)	172 (9)	181 (15)	183 (7)	171 (9)	180 (11)	182 (10)	169 (13)	181 (9)	184 (8)	171 (9)	180 (11)
Weight (median, Q; kg)	88 (13)	66 (12)	80 (21)	86 (12)	71 (16)	81 (16)	82 (13)	70 (11)	80 (13)	86 (12)	69 (14)	81 (17)
Experience^†^ (median, Q; years)	14 (14)	11 (18)	13 (16)	25 (18)	17 (26)	22 (24)	32 (27)	30 (37)	32 (31)	25 (23)	17 (23)	22 (26)

*AB – merged A and B age categories of masters rowers (age 27-42 years); CDE – merged C, D, and E age categories (age 43-59 years); and F+ – merged F to M age categories (age 60 years and older); Q – Interquartile range.

†Experience – period since the beginning of competitive rowing on the international level.

### Statistical methods

Statistical analysis was performed in the same manner as previously published (6,7). Continuous variables are presented as median and interquartile range, and categorical variables as absolute and relative frequencies. The Mann-Whitney test was used for comparisons of continuous variables and the χ^2^ test for categorical variables. When appropriate, the Yates continuity correction was used with χ^2^ tests. The Fisher exact test was used to assess the differences in injury rates between men and women, and the analysis was performed using the OpenEpi module for comparing rates (*http://www.openepi.com/Menu/OpenEpiMenu.htm*). One-sample *t* test was performed to compare the mean injury rate per masters rower per year with those of juniors and seniors obtained from previously published data (the data about injury rates deviated from normality but the large sample size compensated for this). The significance level was *P* < 0.05. All other analyses were performed using IBM SPSS Statistics v. 24 for Windows (IBM, Armonk, NY, USA; licensed to the Faculty of Humanities and Social Sciences, University of Split).

## RESULTS

### Frequency of injuries and injury mechanism

Of 743 masters rowers, 248 (33.4%; 158 men and 90 women) sustained 359 injuries. Some master rowers experienced multiple injuries: 160 experienced 1 injury (55 women and 105 men), 66 experienced 2 injuries (25 women and 41 men), 21 rowers experienced 3 injuries (9 women and 12 men), while 1 woman rower experienced four injuries (Supplementary Table 1(supplementary table 1)).

The vast majority of reported injuries were chronic (Table 2). The mean injury rate per year for all masters rowers was 0.48 injuries per rower (2.25 injuries per 1000 training sessions per rower). There was no significant difference in the injury rate and frequency of either acute or chronic injuries between men and women rowers in any age group (data not shown).

**Table 2:** Acute (traumatic) and chronic (overuse) injuries among masters rowers*

	AB			CDE			F+			All		
Gender	men	women	both	men	women	both	men	women	both	men	women	both
Number of all injuries	44	47	91	74	123	197	18	53	71	136	223	359
Acute injuries												
on water (%)	7 (15.9)	12 (25.5)	19 (20.9)	11 (14.9)	19 (15.5)	30 (15.2)	2 (11.1)	11 (20.8)	13 (18.3)	20 (14.7)	42 (18.8)	62 (17.3)
on ergometer (%)	1 (2.3)	0 (0)	1 (1.1)	2 (2.7)	1 (0.8)	3 (1.5)	1 (5.6)	0 (0)	1 (1.4)	4 (2.9)	1 (0.5)	5 (1.4)
in the gym (%)	1 (2.3)	2 (4.3)	3 (3.3)	2 (2.7)	3 (2.4)	5 (2.5)	1 (5.6)	0 (0)	1 (1.4)	4 (2.9)	5 (2.2)	9 (2.5)
during cross-training (%)	7 (15.9)	6 (12.8)	13 (14.3)	10 (13.5)	24 (19.5)	34 (17.3)	1 (5.6)	11 (20.8)	12 (16.9)	18 (13.3)	41 (18.4)	59 (16.4)
all (%)	16 (36.4)	20 (42.6)	36 (39.6)	25 (33.8)	47 (38.2)	72 (36.5)	5 (27.9)	22 (41.6)	27 (38.0)	46 (33.8)	89 (39.1)	135 (37.6)
Chronic injuries (%)	28 (63.6)	27 (57.4)	55 (60.4)	49 (66.2)	76 (61.8)	125 (63.5)	13 (72.1)	31 (58.4)	44 (62.0)	90 (66.2)	134 (60.1)	224 (62.4)
A/C ratio	1:1.8	1:1.4	1:1.5	1:2.0	1:1.6	1:1.7	1:2.6	1:1.4	1:1.6	1:2.0	1:1.5	1:1.7
MIR	0.41	0.47	0.44	0.54	0.52	0.54	0.40	0.55	0.43	0.47	0.51	0.48
MNI 1000	1.91	2.27	2.07	2.55	2.43	2.50	2.26	1.85	1.94	2.20	2.35	2.25

*AB – merged A and B age categories of masters rowers (age 27-42 years); CDE – merged C, D, and E age categories (age 43-59 years); and F+ – merged F to M age categories (age 60 years and older); A/C – acute/chronic injury; MIR – mean injury rate per year per a rower; and MNI 1000 – mean number of injuries per 1000 training sessions per a rower.

### Anatomical distribution of sustained injuries

The most common site of both acute and chronic injuries was the low back (32.6% of all injuries) (Table 3). It was the most frequently injured localization in all groups: 31.8% of all injuries in AB group, 34.5% in CDE group, and 28.2% in F+ group (Supplementary Table 2(supplementary table 2), Supplementary Table 3(supplementary table 3), and Supplementary Table 4(supplementary table 4)). Interestingly, rowers in F+ group sustained more injuries in the upper body (neck/cervical spine, elbow, lower arm, and wrist) compared with AB and CDE groups (χ^2^ = 17.13, *P* < 0.001). Conversely, rowers in AB group sustained more injuries in the lower body (pelvis/groin/buttock/hip/thigh, knee, and lower leg) compared with CDE and F+ groups (χ^2^ = 24.54, *P* < 0.001).

**Table 3:** Acute and chronic injuries among masters rowers divided by the anatomic region (some rowers reported multiple injuries)

Anatomic region	Acute injuries (%)			Chronic injuries (%)			Total (%)		
	men	women	total	men	women	total	men	women	total
**Head**	0 (0)	0 (0)	0 (0)	0 (0)	0 (0)	0 (0)	0 (0)	0 (0)	0 (0)
**Neck/cervical spine**	2 (2.2)	2 (4.3)	4 (3.0)	4 (3.0)	1 (1.1)	5 (2.2)	6 (2.7)	3 (2.2)	9 (2.5)
**Shoulder/upper arm**	6 (6.7)	4 (8.7)	10 (7.4)	16 (11.9)	12 (13.3)	28 (12.5)	22 (9.9)	16 (11.8)	38 (10.6)
**Elbow**	2 (2.2)	1 (2.2)	3 (2.2)	21 (15.7)	14 (15.6)	35 (15.6)	23 (10.3)	15 (11.0)	38 (10.6)
**Lower arm/wrist**	4 (4.5)	1 (2.2)	5 (3.7)	10 (7.5)	4 (4.4)	14 (6.3)	14 (6.3)	5 (3.7)	19 (5.3)
**Hand**	2 (2.2)	2 (4.3)	4 (3.0)	4 (3.0)	2 (2.2)	6 (2.7)	6 (2.7)	4 (2.9)	10 (2.8)
**Chest/thoracic spine**	3 (3.4)	4 (8.7)	7 (5.2)	5 (3.7)	3 (3.3)	8 (3.6)	8 (3.6)	7 (5.1)	15 (4.2)
**Trunk/abdomen**	0 (0)	1 (2.2)	1 (0.7)	0 (0)	0 (0)	0 (0)	0 (0)	1 (0.7)	1 (0.3)
**Low back**	34 (38.2)	13 (28.3)	47 (34.8)	39 (29.1)	31 (34.4)	70 (31.3)	73 (32.7)	44 (32.4)	117 (32.6)
**Pelvis/groin/buttock/hip/thigh**	12 (13.5)	7 (15.2)	19 (14.1)	13 (9.7)	5 (5.6)	18 (8.0)	25 (11.2)	12 (8.8)	37 (10.3)
**Knee**	13 (14.6)	8 (17.4)	21 (15.6)	15 (11.2)	15 (16.6)	30 (13.4)	28 (12.6)	23 (16.9)	51 (14.2)
**Lower leg**	3 (3.4)	1 (2.2)	4 (3.0)	2 (1.5)	3 (3.3)	5 (2.2)	5 (2.2)	4 (2.9)	9 (2.5)
**Ankle**	8 (9.0)	2 (4.3)	10 (7.4)	1 (0.7)	0 (0)	1 (0.4)	9 (4.0)	2 (1.5)	11 (3.1)
**Foot**	0 (0)	0 (0)	0 (0)	4 (3.0)	0 (0)	4 (1.8)	4 (1.8)	0 (0)	4 (1.1)
**Total**	89 (100)	46 (100)	135 (100)	134 (100)	90 (100)	224 (100)	223 (100)	136 (100)	359 (100)

### Severity of sustained injuries

Sustained injuries most frequently did not lead to loss of training or competition time in any of the groups, ie, they were incidental injuries (Table 4). In AB group, these incidental injuries comprised 35.2% (n = 32) of all injuries, in CDE group 37.1% (n = 73), and in F+ group 46.5% (n = 33) (Supplementary Table 5(supplementary table 5)). The injuries that caused loss of training or competition time were most frequently categorized as moderate injuries, followed by minor injuries. In AB group, major injuries accounted for 10.9% (n = 10) of all injuries, 8 of which were acute and 2 chronic. In CDE group, major injuries accounted for 8.6% (n = 17) of all injuries, 9 acute and 8 chronic, while in F+ group major injuries accounted for 7.0% (n = 5) of all injuries, 4 acute and 1 chronic. One 49-year-old woman rower from CDE group experienced a rib stress fracture (0.1% of all rowers who participated in the study and 0.3% of all rowers in CDE group). The injury was classified as major.

**Table 4:** Severity of injuries among masters rowers, classified according to Morgan and Oberlander (18)

Injured anatomic region	All			
**incident** **N (%)**	**minor** **N (%)**	**moderate N (%)**	**major** **N (%)**
**Head**	0 (0.0)	0 (0.0)	0 (0.0)	0 (0.0)
**Neck/cervical spine**	4 (2.9)	3 (3.8)	2 (1.8)	0 (0.0)
**Shoulder/upper arm**	16 (11.6)	8 (10.3)	11 (9.9)	3 (9.4)
**Elbow**	27 (19.6)	7 (9.0)	4 (3.6)	0 (0.0)
**Lower arm/wrist**	9 (6.5)	4 (5.1)	3 (2.7)	3 (9.4)
**Hand**	7 (5.1)	2 (2.6)	1 (0.9)	0 (0.0)
**Chest/thoracic spine**	1 (0.7)	5 (6.4)	5 (4.5)	4 (12.5)
**Trunk/abdomen**	0 (0.0)	0 (0.0)	1 (0.9)	0 (0.0)
**Low back**	28 (20.3)	27 (34.6)	52 (46.9)	10 (31.2)
**Pelvis/groin/buttock/hip/thigh**	17 (12.3)	8 (10.3)	8 (7.2)	4 (12.5)
**Knee**	21 (15.3)	9 (11.5)	14 (12.6)	7 (21.9)
**Lower leg**	4 (2.9)	2 (2.6)	2 (1.8)	1 (3.1)
**Ankle**	2 (1.4)	3 (3.8)	6 (5.4)	0 (0.0)
**Foot**	2 (1.4)	0 (0.0)	2 (1.8)	0 (0.0)
**Total**	138 (100.0)	78 (100.0)	111 (100.0)	32 (100.0)

### Acute injuries sustained during rowing-specific training

Rowing-specific training in the boat or on rowing ergometer resulted in 20 acute injuries in AB group (55.6% of all acute injuries in this group), 33 acute injuries in CDE group (45.8% of all acute injuries in this group), and 14 acute injuries in F+ group (51.9% of all acute injuries this group) (Tables 2 and 5). Two boat collisions were reported in CDE group, resulting with two moderate low back injuries. No boat collisions resulting in injury were reported in AB and F+ group.

**Table 5:** The most common sites and severity of acute injury sustained during on-water training among masters rowers*

Injury sustained during on-water training	AB	CDE	F+
Injury site			
Low back	10	17	5
Pelvis/groin/buttock/hip/thigh	4	1	3
Chest/thoracic spine	2	2	1
Neck/cervical spine	1	1	1
Shoulder	0	3	0
Lower arm/wrist	0	2	1
Hand	1	1	0
Elbow	0	0	1
Knee	0	0	1
Lower leg	1	0	0
Injury severity			
Incidental	4	2	3
Minor	6	6	5
Moderate	6	17	3
Major	3	3	2

*AB – merged A and B age categories of masters rowers (age 27-42 years); CDE – merged C, D, and E age categories (age 43-59 years); and F+ – merged F to M age categories (age 60 years and older).

The rowing ergometer was used by 89.4% (n = 186) of all rowers in AB group, 85.3% (n = 314) in CDE group, and 85.0% (n = 142) in F+ group. On rowing ergometer, 1 rower in AB group sustained a moderate injury in the low back region. In CDE group, 3 rowers (0.8%) sustained injuries, 2 moderate injuries in the low back and 1 minor injury in the shoulder/upper arm region. In F+ group, 1 rower (0.6%) sustained a moderate injury, ie, the trunk/abdomen region.

There was a significant association between the length of training sequences and presence of acute low back pain in AB group. Rowers who practiced longer sequences had lower incidence of acute low back pain (χ^2^ = 15.665, *P* = 0.004). On the other hand, those who practiced longer sequences on rowing ergometer had significantly more chronic knee injuries in AB group (χ^2^ = 17.144, *P* = 0.002). No such association was observed in older age groups. In addition, average length of training sessions on the ergometer shorter than 30 minutes was associated with higher incidence of low back injuries in group F+ (χ^2^ = 4.114, *P* = 0.043), while there was no such association in AB (χ^2^ = 0.685, *P* = 0.408) and CDE groups (χ^2^ = 0.017, *P* = 0.897).

### Acute injuries acquired in the gym or during cross training

Altogether, 9 acute injuries (6.7% of all acute injuries) were sustained during training in the gym (Table 2). In AB group, 3 minor injuries were localized in the elbow, low back, and pelvis/groin/buttock/hip/thigh region. In CDE group there was 3 moderate (1 in the shoulder and 2 in the low-back region), 1 minor (the low back), and 1 incidental injury (pelvis/groin/buttock/hip/thigh region). In F+ group there was 1 incidental injury in the shoulder region.

Different cross-training modalities were used by 91.8% (n = 191) of rowers in AB group, 84.8% (n = 312) rowers in CDE group, and 70.7% (n = 118) rowers in F+ group. During cross-training, 59 injuries (43.7% of all acute injuries) were sustained (Table 6 and Supplementary Table 6(supplementary table 6)). In AB group, 13 rowers (6.8% of those who use cross training modalities), 6 men and 7 women, sustained 13 acute injuries. In CDE group, 31 rowers (9.9% of those who use cross training modalities), 22 men and 9 women, sustained 34 acute injuries (3 rowers reported 2 acute injuries). In F+ group, 10 rowers (8.5% of those who use cross training modalities), 9 men and 1 woman, sustained 12 injuries (1 rower reported 3 acute injuries). The majority of injuries sustained during cross training were moderate injuries (n = 27), followed by major (n = 13), minor (n = 12), and incidental (n = 7) injuries.

**Table 6:** Number of cross-training and associated acute injuries in masters rowers

Cross training*	Running	Cycling	Nordic skiing	Alpine skiing	Soccer
Practicing rowers	442	345	129	73	9
Injured rowers (%)	25 (5.7)	9 (2.6)	4 (3.1)	6 (8.2)	5 (55.6)
Severity of traumatic injury					
incident	1	1	2	0	1
minor	8	1	0	1	1
moderate	12	4	1	4	2
major	4	3	1	4	1
Cross-training specific injuries	25	9	4	9	5
Most frequent regions affected by more severe injuries among all injuries of that cross-training (%)	knee 10 (40)	hip 2 (22)	thigh 1 (25)	knee 5 (56)	knee 1 (20)

*Volleyball resulted with 3 traumatic injuries, and golf, badminton, gardening, and kayak resulted with 1 traumatic injury each (data not shown).

### Demographic characteristics, rowing experience, training characteristics, and injury occurrence

In all groups there was no association between height, weight, or body mass index and the occurrence rate of acute or chronic injuries (data not shown). A total of 7.0% (n = 52) of the participants competed at the elite level in their youth, ie, they rowed in A final either at senior’s World Rowing Championships or the Olympic Games (30 of them [4.0% of the participants] won a medal at the Championships and 7 at the Olympics [0.9% of the participants]). The majority of the participants (n = 492; 66.2%) previously competed in FISA World Rowing Masters Regatta(s) and 304 (40.9%) won a medal in masters regattas. In all groups men rowers had significantly longer rowing experience in years compared to women rowers (AB: women 17 [0-33] vs men 21 [2-33], *P* = 0.002; CDE: women 26 [1-46] vs men 34 [3-48 ], *P* < 0.001; F+: women 35 [5-70] vs men 46 [2-64], *P* = 0.025, Mann-Whitney test). Interestingly, there was no association between rowing experience and acute or chronic injury occurrence. Rowers in AB group who won a medal at world championships or the Olympic games experienced significantly fewer acute injuries compared with those who did not win a medal (χ^2^ = 4.321, *P* = 0.038), while in CDE and F+ group this was not the case (CDE group: χ^2^ = 0.716, *P* = 0.398; group F+: χ^2^ = 0.035, *P* = 0.851). However, rowers in CDE group who won a medal at U-23 world championships experienced significantly fewer acute injuries compared with those who did not compete at the competition (χ^2^ = 4.264, *P* = 0.039). In F+ group, rowers who won a medal at the Olympic games experienced significantly fewer acute injuries than those who did not win a medal (χ^2^ = 8.328, *P* = 0.004).

Regarding the rowing discipline, scull rowers in group F+ experienced significantly more chronic low back injuries compared to sweep rowers (χ^2^ = 4.973, *P* = 0.026); however, this was not the case in younger age groups.

Engaging in multiple cross-training modalities per rower was related to significantly more rowers sustaining acute injuries in AB group (χ^2^ = 24.751, *P* = 0.001) and CDE group (χ^2^ = 15.278, *P* = 0.033), while in F+ group the difference was not significant (χ^2^ = 6.957, *P* = 0.138). There was no association between the number of different cross-training modalities and rate of chronic injuries in any of the groups.

## DISCUSSION

The present study is the first to investigate injuries in international masters rowers. Although winning a medal at some elite level seniors rowing competition was associated with fewer acute injuries sustained during the master rowing season, there was no association between rowing experience and acute or chronic injury occurrence among masters rowers. The discrepancy might be due to a very small number of masters rowers who won a medal at elite level senior competition. Also, the lack of influence of rowing experience on injury occurrence might be associated with the fact that median of rowing experience was 13 years even in the least experienced group of master rowers, ie AB group, while other groups of masters rowers were even more experienced.

Master rowers had lower annual aggregate injury rate (0.48 injuries per rower) compared to elite level junior (0.99 injuries per rower; one sample *t* test, *t* = -17.6, df = 742, *P* < 0.001) (7) and senior rowers (0.92 injuries per rower; one sample *t* test, *t* = -15.2, df = 742, *P* < 0.001) (6) (Table 7). This indicates that they enjoy “safety” of training and competing in rowing. It is also in line with the study of Dunsky and Netz (19), who found a very low injury rate during physical activity in advanced age compared with other ages.

**Table 7:** Mean injury rate per year per a rower (MIR) and mean number of injuries per 1000 training sessions per a rower (MNI 1000) for junior and senior rowers

Rowers	Men	Women	Total
Junior*			
MIR	0.90	1.10	0.99
MNI 1000	1.95	2.36	2.10
Senior lightweight category^†^			
MIR	0.86	0.86	0.86
MNI 1000	1.69	1.60	1.67
Senior open-weight category^†^			
MIR	0.90	1.02	0.95
MNI 1000	1.72	1.90	1.80
Seniors all^†^			
MIR	0.88	0.98	0.92
MNI 1000	1.71	1.83	1.75

*According to Smoljanovic et al, 2009 (7).

†According to Smoljanovic et al, 2015 (6).

Regular rowing provides a number of health benefits to the masters athlete (8), such as improving serum cholesterol levels (20) and preventing sarcopenia (21) and bone weakening (22). As a non-high-impact sport (10), rowing is suitable for people who underwent total hip (23,24) or knee arthroplasty. These benefits, together with the positive psychological impact of camaraderie inside and outside of the boat, are the reasons why there are so many master rowers worldwide and why master regattas are the largest FISA rowing event.

Rakovac et al (25) found that men and women junior rowers competing at the 2007 World Junior Rowing Championships were significantly taller than rowers who competed at the 1997 World Junior Rowing Championships (men 187.4 vs 188.4 cm and women 174.5 vs 176.6 cm). The finding partially confirmed the Carter’s suggestion that the height and weight of the nationally ranked athletes was increasing by about 2 cm and 5 kg per decade (26). Although the majority of masters rowers included in this research were not elite level rowers in their youth, increase in height was observed over time (Table 1). The height of women rowers in the F+ group was 169 cm, compared with 172 cm in the AB group. The height of men rowers in the F+ group was 182 cm, compared with 187 cm in the AB group.

The reason for the low injury rate among masters rowers might be the smaller number and lower intensity of their training sessions. Rib stress fractures occur in 8.1%-16.4% of elite rowers, 2% of university rowers, and 1% of junior elite rowers (27). Only one of 743 master rowers in our study suffered from rib stress fracture, which may reflect low training intensity in masters athletes. In addition, 93% of the participants had not been elite level rowers in their youth. As regular rowing provides numerous benefits to health and well-being, accompanied with low injury risk, it would be interesting to find out why the majority of elite rowers did not continue training in the masters category after the end of their elite career.

When the number of training sessions was taken into consideration our data revealed that masters rowers (2.25 injuries per 1000 training sessions per rower) were slightly more prone to injuries than younger elite level junior rowers (7) and senior rowers (8) (Table 7). Kammerlander et al (28) also found that older adults (65+) remaining active in sports suffered from more sports-related injuries than their inactive age peers. However, the majority of the injuries experienced by master rowers in our research remained of low severity, ie, they did not cause long-lasting loss of training or competition time.

The most common injury site in masters rowers was the low back, as was the case in junior (7), senior (6,29), and elite (30) rowers, but not in collegiate rowers, in whom the most common injury site was the knee (31). The interesting finding related to the injury distribution in masters rowers was the higher frequency of upper body injuries in F+ group (neck/cervical spine, elbow, lower arm, and wrist). This is in contrast to earlier findings of injury patterns in junior and senior rowers (6,7). It is recognized that people aged 60 and over experience a decrease in strength and balance with aging (32). One could hypothesize that with loss of lower extremity strength, older masters rowers might change their rowing technique, and that increased or more pronounced use of upper extremities might result in a higher frequency of overuse injuries. We also reported more low back injuries in rowers from group F+, whose average length of training sessions on the ergometer shorter than 30 minutes might also be explained by the aging process. Only masters athletes with a better functional capacity might be able to perform training sessions longer than 30 minutes on the ergometer. Masters rowers from F+ group who cannot perform longer training sessions on the ergometer due to the functional decrease in strength and balance are more prone to acute injuries in the low back even when performing shorter rowing distances.

A limitation of the study is the retrospective study design relying on the accuracy of rowers’ reports. Another limitation is that there were master rowers who sustained injuries during the observed rowing season and were not able to recover and participate in the regatta. For these reasons, the incidence of rowing injuries, especially major ones, may be underestimated. However, to our best knowledge this is still the only study of injuries among master rowers.

Master rowers can safely practice their physical activity as the injuries they suffer from in rowing are not severe. Their risk of injury is low and the injuries are mainly located in the low back area. Masters rowers in F+ group should be aware that their upper extremities might be more prone to the injuries than their lower extremities.
