# Supplementary material for: Sport injuries in international masters rowers: a cross-sectional study
Source: Croat Med J. 2018 Oct;59(5):258–66. doi: 10.3325/cmj.2018.59.258 (PMC6240823; doi:10.3325/cmj.2018.59.258)
Supplement: Supplementary Table 1 [file CroatMedJ_59_s001.pdf]

Supplement table 1. Number of injured master rowers and number of injuries sustained by them. Acute (traumatic) and chronic (overuse) injuries among master rowers. AB - merged A and B age categories of master rowers (age 27-42 years), CDE - merged C, D and E age categories of master rowers (age 43-59 years), and F+ – merged F to M age categories of master rowers (age 60 years and older), NoI – number of injured master rowers, 1I – number of master rowers who sustained one injury, 2I – number of master rowers who sustained two injuries, 3I – number of master rowers who sustained three injuries, 4I – number of master rowers who sustained four injuries.

	AB			CDE			F+			All		
Gender	Men	Women	Both	Men	Women	Both	Men	Women	Both	Men	Women	Both
NoI	36	29	65	85	48	133	37	13	50	158	90	248
1I	26	17	43	53	29	82	26	9	35	105	55	160
2I	9	9	18	26	13	39	6	3	9	41	25	66
3I	1	3	4	6	5	11	5	1	6	12	9	21
4I	0	0	0	0	1	1	0	0	0	0	1	1
