# Supplementary material for: Sport injuries in international masters rowers: a cross-sectional study
Source: Croat Med J. 2018 Oct;59(5):258–66. doi: 10.3325/cmj.2018.59.258 (PMC6240823; doi:10.3325/cmj.2018.59.258)
Supplement: Supplementary Table 2 [file CroatMedJ_59_s002.pdf]

Supplement Table 2. Acute and chronic injuries among master rowers belonging to group AB (merged A and B age categories of master rowers (age 27-42 years)), divided by the anatomic region (some rowers reported multiple injuries).

Anatomic region	Acute injuries (%)			Chronic injuries (%)			Total (%)		
	Female	Male	Total	Female	Male	Total	Female	Male	Total
**Head**	0 (0)	0 (0)	0 (0)	0 (0)	0 (0)	0 (0)	0 (0)	0 (0)	0 (0)
**Neck/Cervical spine**	0 (0)	1 (5.0)	1 (2.8)	0 (0)	1 (3.7)	1 (1.8)	0 (0)	2 (4.3)	2 (2.2)
**Shoulder/Upper arm**	0 (0)	0 (0)	0 (0)	3 (10.7)	1 (3.7)	4 (7.3)	3 (6.8)	1 (2.1)	4 (4.4)
**Elbow**	0 (0)	1 (5.0)	1 (2.8)	4 (14.3)	2 (7.5)	6 (10.9)	4 (9.1)	3 (6.4)	7 (7.7)
**Lower arm/Wrist**	0 (0)	1 (5.0)	1 (2.8)	2 (7.1)	1 (3.7)	3 (5.5)	2 (4.6)	2 (4.3)	4 (4.4)
**Hand**	1 (6.2)	1 (5.0)	2 (5.6)	1 (3.6)	1 (3.7)	2 (3.6)	2 (4.6)	2 (4.3)	4 (4.4)
**Chest/Thoracic Spine**	1 (6.2)	1 (5.0)	2 (5.6)	1 (3.6)	1 (3.7)	2 (3.6)	2 (4.6)	2 (4.3)	4 (4.4)
**Trunk/Abdomen**	0 (0)	0 (0)	0 (0)	0 (0)	0 (0)	0 (0)	0 (0)	0 (0)	0 (0)
**Low back**	5 (31.4)	8 (40.0)	13 (36.0)	8 (28.6)	8 (29.6)	16 (29.1)	13 (29.5)	16 (34.0)	29 (31.8)
**Pelvis/Groin/Buttock/Hip/Thigh**	3 (18.8)	4 (20.0)	7 (19.4)	2 (7.1)	3 (11.1)	5 (9.1)	5 (11.3)	7 (14.9)	12 (13.2)
**Knee**	4 (25.0)	2 (10.0)	6 (16.6)	5 (17.9)	8 (29.6)	13 (23.7)	9 (20.4)	10 (21.2)	19 (20.9)
**Lower leg**	1 (6.2)	1 (5.0)	2 (5.6)	2 (7.1)	0 (0)	2 (3.6)	3 (6.8)	1 (2.1)	4 (4.4)
**Ankle**	1 (6.2)	0 (0)	1 (2.8)	0 (0)	0 (0)	0 (0)	1 (2.3)	0 (0)	1 (1.1)
**Foot**	0 (0)	0 (0)	0 (0)	0 (0)	1 (3.7)	1 (1.8)	0 (0)	1 (2.1)	1 (1.1)
**Total**	16 (100.0)	20 (100.0)	36 (100.0)	28 (100.0)	27 (100.0)	55 (100.0)	44 (100.0)	47 (100.0)	91 (100.0)
