# Supplementary material for: Sport injuries in international masters rowers: a cross-sectional study
Source: Croat Med J. 2018 Oct;59(5):258–66. doi: 10.3325/cmj.2018.59.258 (PMC6240823; doi:10.3325/cmj.2018.59.258)
Supplement: Supplementary Table 3 [file CroatMedJ_59_s003.pdf]

Supplement Table 3. Acute and chronic injuries among master rowers belonging to group CDE (merged C, D and E age categories of master rowers (age 43-59 years)), divided by the anatomic region (some rowers reported multiple injuries).

Anatomic region	Acute injuries (%)			Chronic injuries (%)			Total (%)		
	Female	Male	Total	Female	Male	Total	Female	Male	Total
Head	0 (0)	0 (0)	0 (0)	0 (0)	0 (0)	0 (0)	0 (0)	0 (0)	0 (0)
Neck/Cervical spine	1 (4.0)	0 (0)	1 (1.4)	1 (2.0)	1 (1.3)	2 (1.6)	2 (2.7)	1 (0.8)	3 (1.5)
Shoulder/Upper arm	3 (12.0)	5 (10.6)	8 (11.1)	7 (14.3)	10 (13.2)	17 (13.6)	10 (13.5)	15 (12.2)	25 (12.7)
Elbow	0 (0)	0 (0)	0 (0)	9 (18.3)	12 (15.8)	21 (16.8)	9 (12.2)	12 (9.8)	21 (10.7)
Lower arm/Wrist	0 (0)	2 (4.3)	2 (2.8)	2 (4.1)	5 (6.6)	7 (5.6)	2 (2.7)	7 (5.7)	9 (4.6)
Hand	1 (4.0)	1 (2.1)	2 (2.8)	0 (0)	2 (2.6)	2 (1.6)	1 (1.3)	3 (2.4)	4 (2.0)
Chest/Thoracic Spine	3 (12.0)	1 (2.1)	4 (5.5)	2 (4.1)	4 (5.3)	6 (4.8)	5 (6.8)	5 (4.1)	10 (5.1)
Trunk/Abdomen	0 (0)	0 (0)	0 (0)	0 (0)	0 (0)	0 (0)	0 (0)	0 (0)	0 (0)
Low back	8 (32.0)	19 (40.5)	27 (37.5)	17 (34.8)	24 (31.6)	41 (32.8)	25 (33.8)	43 (34.9)	68 (34.5)
Pelvis/Groin/Buttock/Hip/Thigh	4 (16.0)	4 (8.5)	8 (11.1)	2 (4.1)	8 (10.5)	10 (8.0)	6 (8.1)	12 (9.8)	18 (9.1)
Knee	4 (16.0)	8 (17.0)	12 (16.7)	9 (18.3)	5 (6.6)	14 (11.2)	13 (17.6)	13 (10.6)	26 (13.2)
Lower leg	0 (0)	2 (4.3)	2 (2.8)	0 (0)	1 (1.3)	1 (0.8)	0 (0)	3 (2.4)	3 (1.5)
Ankle	1 (4.0)	5 (10.6)	6 (8.3)	0 (0)	1 (1.3)	1 (0.8)	1 (1.3)	6 (4.9)	7 (3.6)
Foot	0 (0)	0 (0)	0 (0)	0 (0)	3 (3.9)	3 (2.4)	0 (0)	3 (2.4)	3 (1.5)
Total	25 (100.0)	47 (100.0)	72 (100.0)	49 (100.0)	76 (100.0)	125 (100.0)	74 (100.0)	123 (100.0)	197 (100.0)
