# Supplementary material for: Sport injuries in international masters rowers: a cross-sectional study
Source: Croat Med J. 2018 Oct;59(5):258–66. doi: 10.3325/cmj.2018.59.258 (PMC6240823; doi:10.3325/cmj.2018.59.258)
Supplement: Supplementary Table 4 [file CroatMedJ_59_s004.pdf]

Supplement Table 4. Acute and chronic injuries among master rowers belonging to group F+ (merged F to M age categories of master rowers (age 60 years and older)), divided by the anatomic region (some rowers reported multiple injuries).

Anatomic region	Acute injuries (%)			Chronic injuries (%)			Total (%)		
	Female	Male	Total	Female	Male	Total	Female	Male	Total
Head	0 (0)	0 (0)	0 (0)	0 (0)	0 (0)	0 (0)	0 (0)	0 (0)	0 (0)
Neck/Cervical spine	1 (20.0)	1 (4.5)	2 (7.4)	0 (0)	2 (6.4)	2 (4.5)	1 (5.6)	3 (5.7)	4 (5.6)
Shoulder/Upper arm	1 (20.0)	1 (4.5)	2 (7.4)	2 (15.4)	5 (16.2)	7 (16.0)	3 (16.6)	6 (11.3)	9 (12.7)
Elbow	1 (20.0)	1 (4.5)	2 (7.4)	1 (7.7)	7 (22.6)	8 (18.2)	2 (11.0)	8 (15.1)	10 (14.0)
Lower arm/Wrist	1 (20.0)	1 (4.5)	2 (7.4)	0 (0)	4 (13.0)	4 (9.1)	1 (5.6)	5 (9.4)	6 (8.5)
Hand	0 (0)	0 (0)	0 (0)	1 (7.7)	1 (3.2)	2 (4.5)	1 (5.6)	1 (1.9)	2 (2.8)
Chest/Thoracic Spine	0 (0)	1 (4.5)	1 (3.7)	0 (0)	0 (0)	0 (0)	0 (0)	1 (1.9)	1 (1.4)
Trunk/Abdomen	1 (20.0)	0 (0)	1 (3.7)	0 (0)	0 (0)	0 (0)	1 (5.6)	0 (0)	1 (1.4)
Low back	0 (0)	7 (31.9)	7 (26.0)	6 (46.1)	7 (22.6)	13 (29.6)	6 (33.2)	14 (26.4)	20 (28.2)
Pelvis/Groin/Buttock/Hip/Thigh	0 (0)	4 (18.2)	4 (14.8)	1 (7.7)	2 (6.4)	3 (6.8)	1 (5.6)	6 (11.3)	7 (9.9)
Knee	0 (0)	3 (13.7)	3 (11.1)	1 (7.7)	2 (6.4)	3 (6.8)	1 (5.6)	5 (9.4)	6 (8.5)
Lower leg	0 (0)	0 (0)	0 (0)	1 (7.7)	1 (3.2)	2 (4.5)	1 (5.6)	1 (1.9)	2 (2.8)
Ankle	0 (0)	3 (13.7)	3 (11.1)	0 (0)	0 (0)	0 (0)	0 (0)	3 (5.7)	3 (4.2)
Foot	0 (0)	0 (0)	0 (0)	0 (0)	0 (0)	0 (0)	0 (0)	0 (0)	0 (0)
Total	5 (100.0)	22 (100.0)	27 (100.0)	13 (100.0)	31 (100.0)	44 (100.0)	18 (100.0)	53 (100.0)	71 (100.0)
