# Supplementary material for: Sport injuries in international masters rowers: a cross-sectional study
Source: Croat Med J. 2018 Oct;59(5):258–66. doi: 10.3325/cmj.2018.59.258 (PMC6240823; doi:10.3325/cmj.2018.59.258)
Supplement: Supplementary Table 5 [file CroatMedJ_59_s005.pdf]

Supplement Table 5. Severity of injuries among master rowers divided by the anatomic region (AB – merged A and B age categories of master rowers (age 27-42 years), CDE – merged C, D and E age categories of master rowers (age 43-59 years), and F+ – merged F to M age categories of master rowers (age 60 years and older)). Classified according to Morgan and Oberlander. (18)

**Category of master rowers**	**AB**				**CDE**				**F+**			
**Injured anatomic region**	**Incident N (%)**	**Minor N (%)**	**Moderate N (%)**	**Major N (%)**	**Incident N (%)**	**Minor N (%)**	**Moderate N (%)**	**Major N (%)**	**Incident N (%)**	**Minor N (%)**	**Moderate N (%)**	**Major N (%)**
**Head**	0 (0.0)	0 (0.0)	0 (0.0)	0 (0.0)	0 (0.0)	0 (0.0)	0 (0.0)	0 (0.0)	0 (0.0)	0 (0.0)	0 (0.0)	0 (0.0)
**Neck/Cervical spine**	1 (3.1)	1 (4.1)	0 (0.0)	0 (0.0)	1 (1.4)	1 (2.6)	1 (1.5)	0 (0.0)	2 (6.1)	1 (6.7)	1 (5.6)	0 (0.0)
**Shoulder/Upper arm**	1 (3.1)	0 (0.0)	3 (12.0)	0 (0.0)	11 (15.1)	5 (12.8)	7 (10.3)	2 (11.8)	4 (12.1)	3 (19.9)	1 (5.6)	1 (20.0)
**Elbow**	3 (9.4)	4 (16.7)	0 (0.0)	0 (0.0)	16 (21.9)	2 (5.1)	3 (4.4)	0 (0.0)	8 (24.3)	1 (6.7)	1 (5.6)	0 (0.0)
**Lower arm/Wrist**	2 (6.3)	0 (0.0)	1 (4.0)	1 (10.0)	4 (5.5)	3 (7.7)	1 (1.5)	1 (5.9)	3 (9.1)	1 (6.7)	1 (5.6)	1 (20.0)
**Hand**	3 (9.4)	0 (0.0)	1 (4.0)	0 (0.0)	3 (4.1)	1 (2.6)	0 (0.0)	0 (0.0)	1 (3.0)	1 (6.7)	0 (0.0)	0 (0.0)
**Chest/Thoracic Spine**	0 (0.0)	3 (12.5)	1 (4.0)	0 (0.0)	1 (1.4)	1 (2.6)	4 (5.9)	4 (23.5)	0 (0.0)	1 (6.7)	0 (0.0)	0 (0.0)
**Trunk/Abdomen**	0 (0.0)	0 (0.0)	0 (0.0)	0 (0.0)	0 (0.0)	0 (0.0)	0 (0.0)	0 (0.0)	0 (0.0)	0 (0.0)	1 (5.6)	0 (0.0)
**Low back**	6 (18.7)	7 (29.2)	13 (52.0)	3 (30.0)	17 (23.2)	14 (35.8)	32 (47.0)	5 (29.3)	5 (15.2)	6 (39.9)	7 (38.8)	2 (40.0)
**Pelvis/Groin/Buttock/Hip/Thigh**	4 (12.5)	4 (16.7)	2 (8.0)	2 (20.0)	9 (12.3)	3 (7.7)	4 (5.9)	2 (11.8)	4 (12.1)	1 (6.7)	2 (11.0)	0 (0.0)
**Knee**	8 (25.0)	4 (16.7)	3 (12.0)	4 (40.0)	9 (12.3)	5 (12.8)	10 (14.7)	2 (11.8)	4 (12.1)	0 (0.0)	1 (5.6)	1 (20.0)
**Lower leg**	3 (9.4)	1 (4.1)	0 (0.0)	0 (0.0)	0 (0.0)	1 (2.6)	1 (1.5)	1 (5.9)	1 (3.0)	0 (0.0)	1 (5.6)	0 (0.0)
**Ankle**	0 (0.0)	0 (0.0)	1 (4.0)	0 (0.0)	1 (1.4)	3 (7.7)	3 (4.4)	0 (0.0)	1 (3.0)	0 (0.0)	2 (11.0)	0 (0.0)
**Foot**	1 (3.1)	0 (0.0)	0 (0.0)	0 (0.0)	1 (1.4)	0 (0.0)	2 (2.9)	0 (0.0)	0 (0.0)	0 (0.0)	0 (0.0)	0 (0.0)
**Total**	32 (100.0)	24 (100.0)	25 (100.0)	10 (100.0)	73 (100.0)	39 (100.0)	68 (100.0)	17 (100.0)	33 (100.0)	15 (100.0)	18 (100.0)	5 (100.0)

(18) Morgan BE, Obrlander MA. An examination of injuries in major league soccer. The inaugural season. Am J Sports Med. 2001;29:426-30.
