# Supplementary material for: Sport injuries in international masters rowers: a cross-sectional study
Source: Croat Med J. 2018 Oct;59(5):258–66. doi: 10.3325/cmj.2018.59.258 (PMC6240823; doi:10.3325/cmj.2018.59.258)
Supplement: Supplementary Table 6 [file CroatMedJ_59_s006.pdf]

Supplement Table 6. Cross-training and associated acute injuries in master rowers. AB - merged A and B age categories of master rowers (age 27-42 years), CDE - merged C, D and E age categories of master rowers (age 43-59 years), and F+ – merged F to M age categories of master rowers (age 60 years and older). * Volleyball resulted with 2 traumatic injuries and golf, gardening and kayak resulted with 1 traumatic injury each (data not shown); † 1 rower in each group reported one additional cross-training injury that does not belong to that cross-training (data not shown).

**AB**						
**Cross training**		**Running**	**Cycling**	**Alpine Skiing**	**Volleyball**	**Soccer**
Practicing rowers		155	108	22	2	1
Injured rowers		7 (4.5%)	2 (1.9%)	2 (9.1%)	1 (50.0%)	1 (100.0%)
Severity of traumatic injury	Incident	1	0	0	1	0
	Minor	2	0	0	0	0
	Moderate	3	1	0	0	0
	Major	1	1	2	0	1
Number of cross-training specific injuries		7	2	2	1	1
Most frequent region affected by more severe injuries among all injuries of that cross-training		Knee 3 (43%)	Lower arm 1 (50%)	Knee 2 (100%)	Hand 1 (100%)	Knee 1 (100%)

**CDE***						
**Cross training**		**Running**	**Cycling**	**Nordic Skiing**	**Alpine Skiing**	**Soccer**
Practicing rowers		222	171	69	39	8
Injured rowers		14 (6.3%)†	4 (2.3%)	3 (4.3%)†	3 (7.7%)	4 (50%)
Severity of traumatic injury	Incident	0	1	1	0	1
	Minor	5	0	0	1	1
	Moderate	7	2	1	1	2
	Major	2	1	1	2	0

Number of cross-training specific injuries	14	4	3	4	4
Most frequent region affected by more severe injuries among all injuries of that cross-training	Knee 6 (43%)	Chest 1 (25%)	Thigh 1 (33%)	Knee 2 (50%)	Low back 1 (25%)

**F+**						
**Cross training**		**Running**	**Cycling**	**Nordic Skiing**	**Alpine Skiing**	**Badminton**
Practicing rowers		65	66	25	12	1
Injured rowers		4 (6.2%)	3 (4.5%)	1 (4.0%)	1 (8.3%)	1 (100.0%)
Severity of traumatic injury	Incident	0	0	1	0	0
	Minor	1	1	0	0	0
	Moderate	2	1	0	3	1
	Major	1	1	0	0	0
Number of cross-training specific injuries		4	3	1	3	1
Most frequent region affected by more severe injuries among all injuries of that cross-training		Ankle 2 (50%)	Lower arm 1 (33%)	Ankle 1 (100%)	Shoulder 1 (33%)	Elbow 1 (100%)
